# Suppression of superconductivity and structural phase transitions under pressure in tetragonal FeS

**DOI:** 10.1038/srep31077

**Published:** 2016-08-08

**Authors:** Xiaofang Lai, Ying Liu, Xujie Lü, Sijia Zhang, Kejun Bu, Changqing Jin, Hui Zhang, Jianhua Lin, Fuqiang Huang

**Affiliations:** 1 Beijing National Laboratory for Molecular Sciences and State Key Laboratory of Rare Earth Materials Chemistry and Applications, College of Chemistry and Molecular Engineering, Peking University, Beijing 100871, China; 2 Beijing National Laboratory for Condensed Matter Physics and Institute of Physics, Chinese Academy of Sciences, Beijing 100190, China; 3 Earth and Environmental Sciences Division and Materials Physics and Applications Division, Los Alamos National Laboratory, Los Alamos, NM 87545, United States; 4 CAS Key Laboratory of Materials for Energy Conversion and State Key Laboratory of High Performance Ceramics and Superfine Microstructure, Shanghai Institute of Ceramics, Chinese Academy of Sciences, Shanghai 200050, China; 5 Collaborative Innovation Center of Quantum Matter, Beijing, China

## Abstract

Pressure is a powerful tool to study iron-based superconductors. Here, we report systematic high-pressure transport and structural characterizations of the newly discovered superconductor FeS. It is found that superconductor FeS (tetragonal) partly transforms to a hexagonal structure at 0.4 GPa, and then completely transforms to an orthorhombic phase at 7.4 GPa and finally to a monoclinic phase above 9.0 GPa. The superconducting transition temperature of tetragonal FeS was gradually depressed by pressure, different from the case in tetragonal FeSe. With pressure increasing, the S-Fe-S angles only slightly change but the anion height deviates farther from 1.38 Å. This change of anion height, together with the structural instability under pressure, should be closely related to the suppression of superconductivity. We also observed an anomalous metal-semiconductor transition at 6.0 GPa and an unusual increased resistance with further compression above 9.6 GPa. The former can be ascribed to the tetragonal-orthorhombic structural phase transition, and the latter to the electronic structure changes of the high-pressure monoclinic phase. Finally, a phase diagram of tetragonal FeS as functions of pressure and temperature was mapped out for the first time, which will shed new light on understanding of the structure and physics of the superconducting FeS.

Iron-based superconductors are of great interest and fundamental importance due to their rich structural and physical properties. Up to now, at least four structure types of iron-based superconductors have been developed, such as ZrCuSiAs-type LnOFeAs (Ln = rare earth metal) and *AeFFeAs* (*Ae* = alkaline metal), ThCr_2_Si_2_-type *Ae*Fe_2_As_2_ and *A*_*x*_Fe_2−y_Se_2_ (A = alkali metal), PbFCl-type AFeAs and anti-PbO-type FeSe^1^,^2^,^3^,^4^,^5^,^6^,^7^. Among them, the Fe_2_*X*_2_ (*X* = pnictogen or chalcogen) layers built up by edge-sharing Fe*X*_4_ tetrahedra are responsible for superconductivity. Their parent compounds usually exhibit a tetragonal to orthorhombic structural transition as well as a paramagnetic to antiferromagnetic transition at low temperatures^8^,^9^, and superconductivity emerges when the structural distortion and magnetic ordering are suppressed by chemical doping or external pressure^10^,^11^. Compared with chemical doping, pressure is a ‘cleaner’ way to tune the crystal structure and physical properties of iron-based superconductors without introducing impurities.

In the iron-based superconductors, the response of the superconducting transition temperature (*T*_*c*_) to pressure is complex and sensitively depends on the composition of the materials and their doping level. For iron pnictides, *dT*_*c*_*/dP* is positive for underdoped compounds and negative for the overdoped ones^12^,^13^. First-principle calculations suggest that this may be caused by the increase of orbital degeneracy in the underdoped regime and decrease of orbital degeneracy at the overdoped regions under pressure^14^. For *A*_*x*_Fe_2−y_Se_2_, it is interesting that a second superconducting phase suddenly reemerges above 11.5 GPa, after the *T*_*c*_ drops from the first maximum of 32 K at 1 GPa^15^. The strongest pressure effect was found in FeSe, whose *T*_*c*_ could be dramatically enhanced from 8 K to 36.7 K under external pressure at 8.9 GPa^16^. ^77^Se NMR (nuclear magnetic resonance) measurement reveals that this may be positively related to the strongly enhanced antiferromagnetic spin fluctuations under high pressure^17^. Moreover, it is found the anion height (the distance of the anion from the Fe plane), rather than the geometry of FeSe_4_ tetrahedra, is the key factor determining *T*_*c*_ of FeSe^18^,^19^, in contrast to the iron pnictide superconductors for which *T*_*c*_ attains a maximum value when the FeAs_4_ tetrahedra form a regular shape and meanwhile the anion height dependence of *T*_*c*_ shows a symmetric curve with a peak around 1.38 Å^20^,^21^,^22^. However, more iron chalcogenide superconductors are needed to verify this issue.

Recently, the discovery of superconductivity in tetragonal FeS^23^, obtained by a low-temperature hydrothermal route similar to that used for producing superconductor Li_1−x_Fe_x_OHFeSe^24^,^25^,^26^, has raised considerable interest. Although its *T*_*c*_ is only 5 K, FeS provides a new and simple platform to realize high-temperature superconductors and to study the underlying mechanism of iron-based superconductivity. According to the low-temperature X-ray diffraction investigations, superconducting FeS does not undergo any structural phase transition on cooling^27^, in contrast to FeSe that shows a structural phase transition from tetragonal to orthorhombic at 70~90 K^28^,^29^. Given the similar crystal and electronic structures between FeS and FeSe^30^, it is of fundamental importance to know whether the strong pressure effect still exists in FeS and how the pressure-tuned superconductivity of FeS relates to its structural properties at atomic level. Here, the behavior of superconductor FeS under pressure was systematically investigated using a combination of electrical resistance, Raman spectroscopy and synchrotron X-ray diffraction (XRD) measurements.

## Results

**Figure 1:**
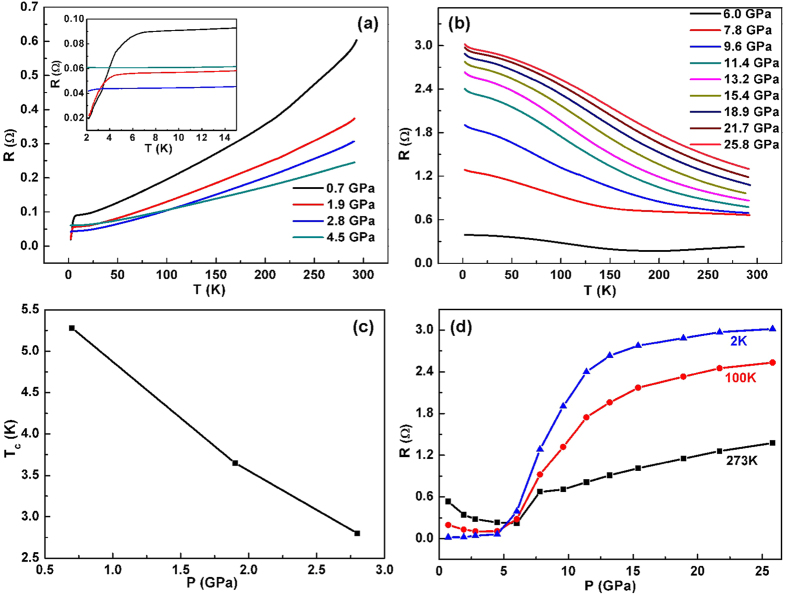
(**a,b**) Temperature dependence of resistance for tetragonal FeS at various pressures, demonstrating a metal-insulator transition at 6.0 GPa. (**c**) Pressure dependence of the superconducting transition temperature *T*_c_. (**d**) Selected pressure dependence of resistance at various temperatures, showing a phase transition at around 6.0 GPa.

At ambient pressure, tetragonal FeS shows metallic behavior with a resistance that decreases with decreasing temperature; when the temperature is below 5 K, it enters into a superconducting state, consistent with the magnetization measurements (Supplementary Fig. S1). Figure 1 shows the electrical properties of tetragonal FeS under pressure. Upon increasing pressure, the *T*_*c*_ of tetragonal FeS gradually drops and the overall resistance decreases until the pressure reaches 6.0 GPa. The pressure-induced resistance drop below 6.0 GPa is associated with the broadening of bands, caused by the shortening and bending of bonds^31^,^32^. It should be noted that the decrease of resistance with pressure was also observed for non-superconducting tetragonal FeS in which neither metallization nor superconductivity was induced by pressure^33^. The low-temperature resistance at 4.5 GPa is a little higher than that at 1.9 and 2.8 GPa and the reason is not clear yet. One possibility is that there exist other phases with higher resistance, as revealed by synchrotron XRD in the following. Obviously, a metal-semiconductor transition occurs at 6.0 GPa, above which the sample becomes semiconducting. As discussed below, this should be associated with a structural phase transition. The pressure dependence of resistance at 2 K, 100 K and 273 K shows that the resistance first decreases with pressure and then increases sharply upon further compression from 6 to 12 GPa, which also implies a phase transition at ~6.0 GPa. It is unusual that, for pressure above 9.6 GPa, the resistance further increases with compression. Since no structural phase transitions occur in the pressure range, this phenomenon should be attributed to electronic structure changes. As shown in Supplementary Fig. S2, when pressure is released, the sample exhibits metallic behavior at 2.6 GPa but insulating behavior below 1.8 GPa; superconductivity is not recovered even at 0.4 GPa. These results suggest that the pressure-induced changes of electrical properties in tetragonal FeS are irreversible.

**Figure 2:**
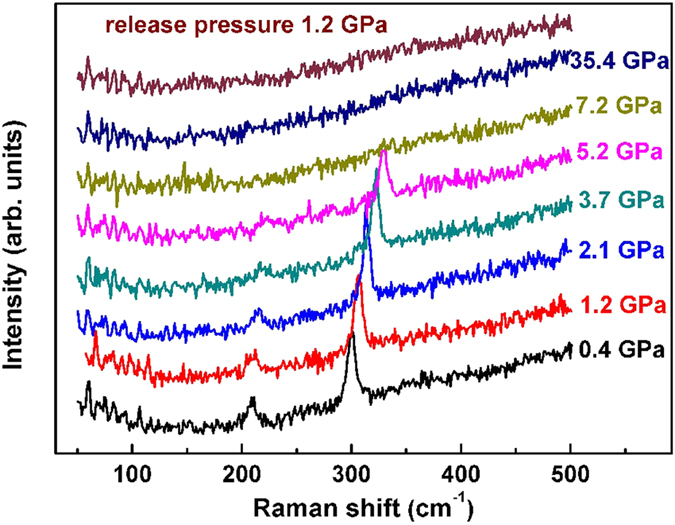
Raman spectra of tetragonal FeS at various pressures. It shows that tetragonal FeS is absent above 7.2 GPa.

Raman spectra of tetragonal FeS collected up to 35.4 GPa at room temperature are shown in Fig. 2. The frequencies of the two well-resolved features seen at 0.4 GPa are 209 and 300 cm^−1^, close to those observed at ambient pressure^34^,^35^. For clarity, we denote these two modes as M1 and M2, which may correspond respectively to one lattice mode of FeS and the symmetric stretching mode^34^,^35^. Upon increasing pressure, the positions of both modes shift to a high energy, with M1 mode hardening by 16.1 cm^−1^ and M2 mode hardening by 29.4 cm^−1^ at 5.2 GPa. At 7.2 GPa, both modes disappear. Generally speaking, vanishing of a Raman peak can be accounted for in two ways: (1) a structural transition where no Raman mode is allowed by symmetry, (2) a transition to a metallic state where the Raman signal becomes extremely weak due to the limited penetration depth of the exciting laser. In this case, our synchrotron XRD results clearly show that the vanishing of the Raman peaks is due to the absence of the tetragonal phase. This structural change is irreversible as gradual depressurization to 1.2 GPa does not lead to reemergence of the characteristic Raman peaks of tetragonal FeS.

**Figure 3:**
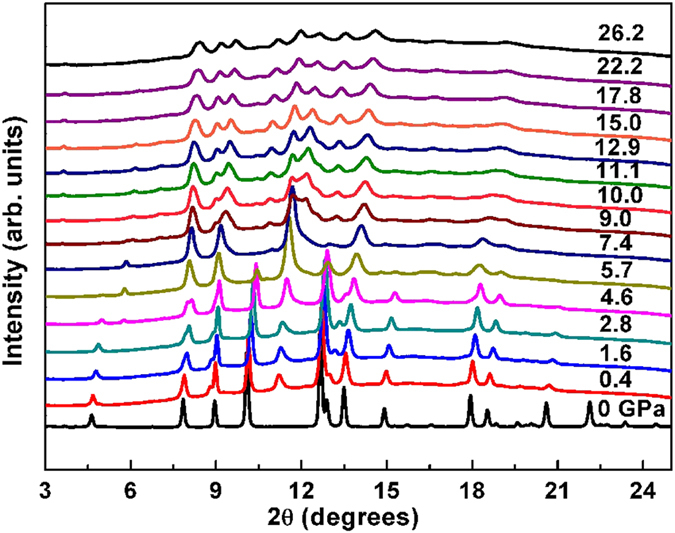
Room-temperature synchrotron XRD patterns of tetragonal FeS at various pressures. Tetragonal FeS first transforms to a hexagonal structure at 0.4 GPa and then to an orthorhombic structure at 4.6 GPa and finally to a monoclinic structure above 9.0 GPa.

Stoichiometric FeS can form tetragonal (space group *P*4*/nmm*) or hexagonal troilite-type structure (space group *P*-62*c*) at ambient pressure and room temperature. A plenty of experimental investigations on high-pressure structures of troilite have been reported, which shows that at both room temperature^36^,^37^ and 17 K^38^, troilite first transforms to an orthorhombic MnP-type structure (space group Pnma) at 3 GPa and then to a monoclinic structure (space group *P*2_1_/*a*) at 7 GPa. Interestingly, semiconductor-metal-semiconductor transitions were observed for troilite under pressure, which is believed to be driven by the above successive structural phase transitions^39^. However, it remains controversial whether the orthorhombic MnP-type FeS is metallic throughout the whole pressure range or not, since semiconducting behavior was reported for FeS at 6 GPa^40^. Here we present the pressure effect on the structure of tetragonal FeS. Synchrotron XRD data of tetragonal FeS at high pressures up to 26.2 GPa at room temperature are shown in Fig. 3. All the observed diffraction reflections obtained at ambient pressure can be indexed with a tetragonal structure. With the onset of pressure, taking *P* = 0.4 GPa for example, new diffraction peaks appear, with the one in regions of 2*θ* between 10.5° and 12° the most prominent. This signals the appearance of the hexagonal structure. Actually, a pressure-induced irreversible tetragonal-hexagonal phase transition has been observed in the non-superconducting tetragonal FeS (the maximum pressure applied is 1.64 GPa) but has not caused much attention^33^. For 0 GPa < *P* < 4.6 GPa, the relative diffraction intensity of the tetragonal and hexagonal structures only slightly change. Then an orthorhombic phase arises at *P* = 4.6 GPa, with the characteristic diffraction peak at 2*θ* = 5.7°. This is in accord with that observed for nanocrystalline mackinawite in which an irreversible first-order structural phase transition from tetragonal to orthorhombic takes place at 3~3.7 GPa, depending on the particle size^41^. As can be seen, the tetragonal phase disappears at 7.4 GPa, consistent with the Raman results. A further structural phase transition to a monoclinic phase begins at 9.0 GPa, above which no more structural changes can be observed. During decompression (Supplementary Fig. S3), the monoclinic phase transforms back to orthorhombic phase at 4.0 GPa and to hexagonal phase at 0 GPa. It is worth noting that tetragonal phase is not recovered. At this point, we reexamined the transport properties shown in Fig. 1 and Supplementary Fig. S2 and found that the hexagonal phase is a semiconductor with extremely high resistance, much higher than the insulating monoclinic phase, and confirmed that the orthorhombic phase is not metallic throughout the whole pressure range. For example, the orthorhombic phase is metallic at 2.6 GPa but semiconducting at 4.6 GPa. This reminds us of the pressure-induced metal-semiconductor transition in troilite which should be closely related to the high-spin to low-spin transition rather than the orthorhombic-monoclinic transition from the point of view of their critical pressures^37^,^39^,^40^,^42^,^43^. Moreover, high-pressure-temperature studies of troilite showed that, for the high-pressure phase between 3~7 GPa, there existed an electronic transition, probably high-spin to low-spin transition, which should not be related to any structural phase transition^44^,^45^. Therefore, we presume that the metal-semiconductor transition of the orthorhombic phase observed in this study may be related to a pressure-induced high-spin to low-spin transition. More measurements, such as Mössbauer spectroscopy and synchrotron X-ray emission spectroscopy are needed to further clarify the nature of this transition.

## Discussion

**Figure 4:**
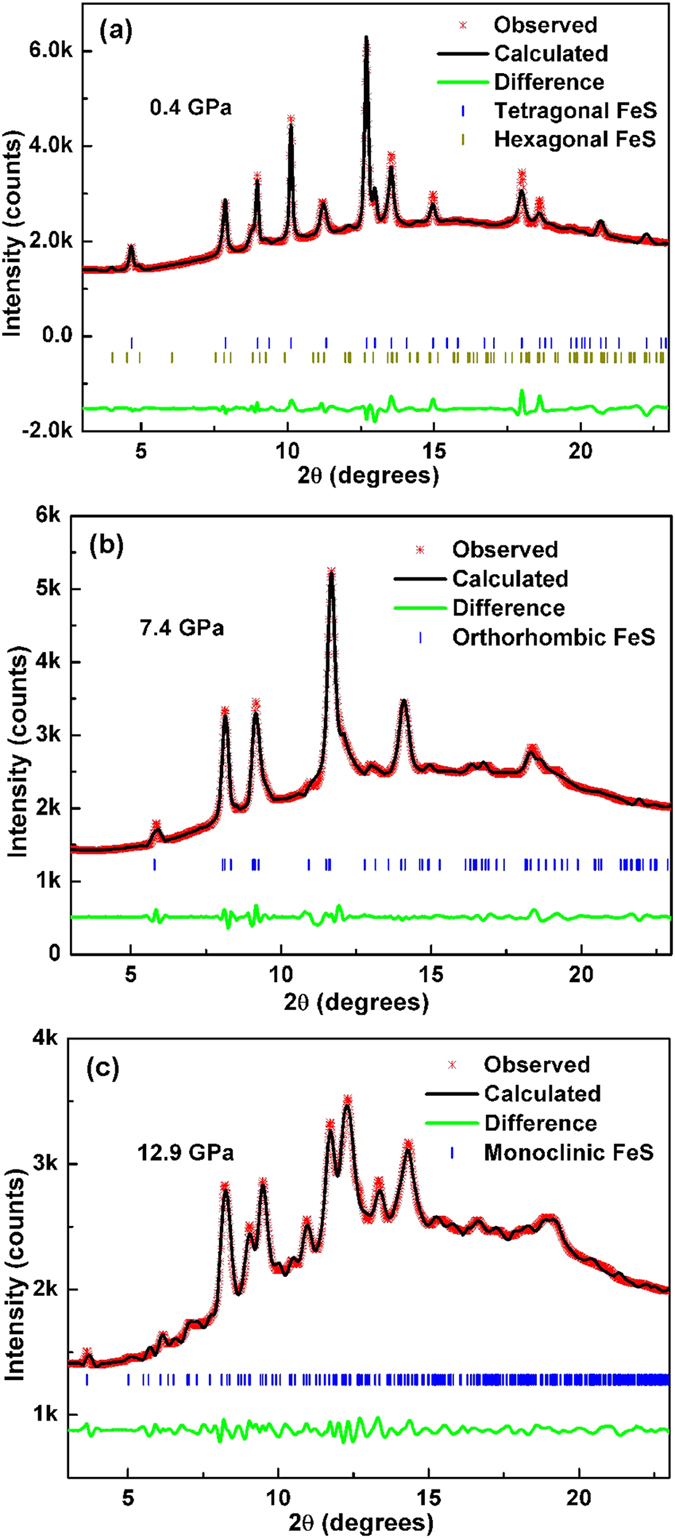
Rietveld refinements of the synchrotron XRD patterns of tetragonal FeS at (**a**) 0.4 GPa, (**b**) 7.4 GPa, and (**c**) 12.9 GPa. Red small points represent the experimental values, black solid line is the calculated pattern, green solid line is the difference between the experimental and calculated values, and blue or dark yellow vertical bars are the Bragg positions.

**Figure 5:**
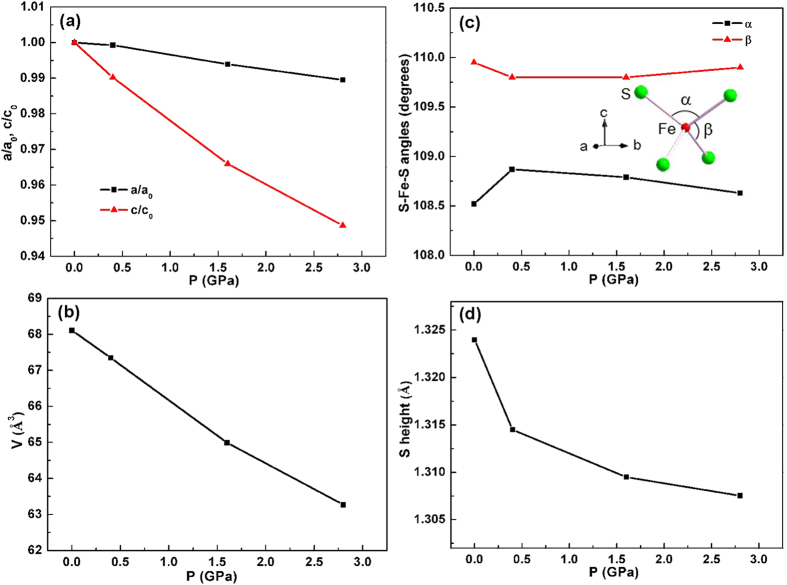
Structural parameters of the tetragonal phase under pressure. (**a**) Pressure dependence of the lattice constants normalized to the ambient-pressure values. (**b**–**d**) Pressure dependence of the unit cell volume, S-Fe-S bond angles and the S height from the iron layer, respectively.

The lattice parameters and volume of tetragonal FeS for the whole pressure range studied, determined by indexing the synchrotron XRD patterns, are shown in Supplementary Fig. S4. To gain more structural information, Rietveld refinements were performed against the diffraction data collected at ambient pressure and at pressures of 0.4, 1.6, 2.8, 7.4 and 12.9 GPa. Representative refinement profiles and refined structure parameters were displayed in Fig. 4 and Supplementary Table SI. These results confirm that the sample is a mixture of tetragonal and hexagonal phases at low pressures and the two high-pressure phases are orthorhombic and monoclinic, respectively. The evolution of the structural parameters of the tetragonal phase (superconducting phase) with pressure is of particular interest because it is closely correlated with superconductivity. Figure 5(a) shows the pressure dependence of the lattice constants of the tetragonal phase, normalized to the ambient-pressure values. The *c* axis is more compressible than *a* axis, with the former contracting by 5% and the latter contracting by 1% at 2.8 GPa. Similar anisotropic compressibility was also observed in FeSe superconductor, which can be accounted for by the anisotropy in bonding of this type of structure^18^. Correspondingly, a remarkable reduction in the unit-cell volume was observed, with 7% reduction at 2.8 GPa. It is well known that the maximum *T*_*c*_ value of iron pnictide superconductors is apparently attained when the FeAs_4_ tetrahedron assumes a regular shape^20^,^22^. However, this rule is not applicable to FeS. On one hand, FeS_4_ tetrahedron is more regular than FeSe_4_ tetrahedron, but FeS has lower *T*_*c*_ than FeSe. On the other hand, upon compression, the S-Fe-S angles of FeS_4_ tetrahedron only slightly change (Fig. 5c), while *T*_*c*_ decreases significantly. As shown in Fig. 5(d), the ambient-pressure S height (anion height) is 1.324 Å and it monotonically decreases with increasing pressure. We know that for both iron pnictides and FeSe, optimal *T*_*c*_ is obtained when the anion height is close to 1.38 Å^18^,^19^,^21^,^22^. Here, it seems also applicable to FeS, that is, *T*_*c*_ decreases as S height deviates farther from 1.38 Å. Therefore, our results support the proposition that the anion height, rather the geometry of FeX_4_ tetrahedra, is the key factor determining *T*_*c*_ of iron-based superconductors containing various anions^19^.

**Figure 6:**
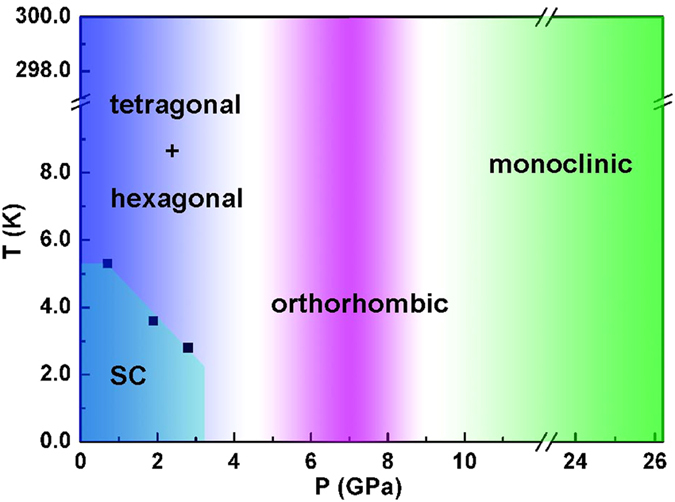
Phase diagram of tetragonal FeS as functions of pressure and temperature. At ambient pressure, the sample is tetragonal (*P*4/*nmm*); once the pressure is onset, the sample becomes a mixture of tetragonal (*P*4/*nmm*) and hexagonal (*P*-62*c*) phases; at intermediate pressure region, the sample is orthorhombic (*Pnma*); at high pressure region, the sample is monoclinic (*P*2_1_/*a*). The solid squares represent *T*_c_ extracted from electrical resistance measurements.

The high-pressure structural phase transitions in tetragonal FeS and troilite are similar except that there exists a tetragonal phase in tetragonal FeS at low pressures. According to the low-temperature X-ray diffraction investigations, the tetragonal phase does not undergo any structural phase transition on cooling at ambient pressure^27^. Actually, at high pressures, the tetragonal phase would not present structural phase transitions on cooling either; otherwise, we could not observe superconductivity at low temperature. The high-pressure studies of troilite reveal that the pressure-induced structural phase transitions of troilite are the same at room temperature and 17 K^36^,^37^,^38^, that is, at a certain high pressure, all the hexagonal, orthorhombic and monoclinic phases would not exhibit structural phase transitions upon cooling. Based on these results, it is reasonable for us to map out a pressure-temperature (*P-T*) phase diagram shown in Fig. 6. This *P-T* phase diagram shares some similarity to that of FeSe which starts to transform to a hexagonal NiAs-type structure at 9.0 GPa and then to an orthorhombic MnP-type structure at higher pressures^18^. Combining with the high-pressure studies on troilite^39^,^40^, we can make a better understanding of the *P-T* phase diagram mapped out here: the tetragonal phase is metallic and the hexagonal phase is semiconducting with strong electronic correlation, both tending to have better electrical conductivity under pressure; the orthorhombic phase indeed shows metallic behavior at low pressures as reported in the literature but it becomes semiconducting at high pressures; the monoclinic phase is a band insulator whose band gap opens up between the nonbonding and antibonding bands, and because the gap increases more rapidly with pressure than the bandwidth, it becomes more insulating with increasing pressure. Based on this *P-T* phase diagram, we can ascribe the observed metal-semiconductor transition in Fig. 1 to the tetragonal-orthorhombic structural phase transition and the increased resistance with pressure above 9.6 GPa to the electronic structure changes of the monoclinic phase.

Comparatively speaking, tetragonal FeS is less stable under pressure than the other iron-based superconductors. Although pressure-induced structural phase transitions were also observed in iron pnictide superconductors, the phase transitions are commonly tetragonal to collapsed tetragonal and isostructural in nature. For example, isostructural phase transitions were reported in ZrCuSiAs-type Nd(O_0.88_F_0.12_)FeAs, ThCr_2_Si_2_-type CaFe_2_As_2_ and EuFe_2_As_2_, and PbFCl-type Na_1−x_FeAs^22^,^46^,^47^,^48^. In these cases, the high-pressure tetragonal phase also possesses superconductivity and provides additional information to study the relationship between superconductivity and atomic crystal structure. For FeSe and FeSe_0.57_Te_0.43_, the pressure onset of structural phase transition is much higher than FeS, 9.0 GPa and 3.0 GPa, respectively^18^,^49^. It is suggested that much higher *T*_*c*_ should be realized by suppressing both the pressure-induced competing tetragonal to hexagonal and orthorhombic to monoclinic structural instabilities in FeSe and FeSe_0.57_Te_0.43_^49^. So apart from the anion height change, the complex structural instabilities of FeS may also be one of the possible reasons responsible for the suppression of superconductivity under pressure.

In conclusion, we have presented systematic high-pressure study on the superconductor FeS. Once the pressure is onset, part of tetragonal FeS will transform to a hexagonal structure. At higher pressures, another two successive phase transitions take place, with the sample being solely orthorhombic at 7.4 GPa and solely monoclinic above 9.0 GPa. The superconductivity of tetragonal FeS is gradually suppressed by pressure, probably due to the S height becoming farther from 1.38 Å and the structural instability under pressure. Thereafter, a metal-semiconductor transition, driven by the tetragonal-orthorhombic phase transition, was observed at 6.0 GPa. The unusual increase of electrical resistance with pressure above 9.6 GPa was attributed to the electronic structure changes of the high-pressure monoclinic phase^39^. Both the structural transitions and the anion height dependence of superconductivity under pressure resemble those observed for FeSe superconductor^18^,^19^, emphasizing the importance of anion height and structural stability, rather than the geometry of FeX_4_ tetrahedra, on superconductivity. Our results will shed new light on understanding of the superconducting mechanism and structure-property relationship of iron chalcogenide superconductors.

## Methods

### Sample synthesis and characterization

Tetragonal FeS was synthesized by the hydrothermal reaction of iron powder with sulfide solution, as described in ref. 23. The ambient-pressure superconducting properties were studied by electrical resistance and magnetic susceptibility measurements in a physical property measurement system (PPMS) of Quantum Design.

### High-pressure electrical resistance measurements

High-pressure electrical resistance measurements were carried out in a diamond anvil cell (DAC) made of CuBe alloy^50^,^51^,^52^,^53^,^54^. A T301 stainless steel gasket was preindented from a thickness of 250 μm to 40 μm, and a hole was drilled at the center with a diameter of about 200 μm. Fine cubic boron nitride (cBN) powder was used to cover the gasket to protect the electrode leads from the metallic gasket. The cBN powder was pressed and further drilled to give a center chamber of 100 μm in diameter. FeS flake with a dimension of 80 μm × 80 μm × 10 μm was loaded with soft NaCl fine powder surrounding it as pressure transmitting medium that can provide a good quasi-hydrostatic pressure environment. We used slim Au wire of 18 μm in diameter as electrodes. Pressure was measured via the ruby fluorescence method^55^ at room temperature before and after each cooling. The DAC was placed inside a MagLab system to perform the experiments with an automatic temperature control. A thermometer located near the diamond of the DAC is used for monitoring the sample temperature.

### High-pressure laser Raman spectroscopy

High-pressure Raman experiments were conducted on FeS flake using Renishaw in Via Raman microscope with laser wavelength of 532 nm and spectral resolution of ∼0.2 cm^−1^. The gasket was T301 stainless steel, which was preindented from a thickness of 250 μm to 40 μm and drilled to give a center hole of 120 μm in diameter. Ruby spheres were pressure monitors.

### High-pressure synchrotron XRD experiments

High-pressure angle-dispersive XRD experiments were carried out at the 16 ID-B station of the High Pressure Collaborative Access Team (HPCAT) at the Advanced Photon Source (APS), Argonne National Laboratory (ANL) using a wavelength of 0.406626 Å. A symmetrical diamond anvil cell was employed to generate high pressure. A rhenium gasket was preindented to 40 μm in thickness followed by laser-drilling the central part to form a 200 μm diameter hole to serve as the sample chamber. The FeS sample and two small ruby balls were loaded into the chamber. Neon was used as the pressure-transmitting medium and the pressure was measured using a ruby fluorescence calibrate^55^. XRD images were collected with a Pilatus detector and were integrated using the Fit2D program^56^. The integrated XRD patterns were further analyzed using the Fullprof program package^57^ for the Rietveld refinements.

## Additional Information

**How to cite this article**: Lai, X. *et al*. Suppression of superconductivity and structural phase transitions under pressure in tetragonal FeS. *Sci. Rep.* **6**, 31077; doi: 10.1038/srep31077 (2016).

## Supplementary Material

Supplementary Information
