# Supplementary material for: Suppression of superconductivity and structural phase transitions under pressure in tetragonal FeS
Source: Sci Rep. 2016 Aug 8;6:31077. doi: 10.1038/srep31077 (PMC4976363; doi:10.1038/srep31077)
Supplement: Supplementary Information [file srep31077-s1.pdf]

Suppression of superconductivity and structural phase transitions under pressure in tetragonal FeS

Xiaofang Lai[1*], Ying Liu[2*], Xujie Lü[3], Sijia Zhang[2], Kejun Bu[4], Changqing Jin[2,5], Hui Zhang[4], Jianhua Lin[1] & Fuqiang Huang[1,4]

[1]Beijing National Laboratory for Molecular Sciences and State Key Laboratory of Rare Earth Materials Chemistry and Applications, College of Chemistry and Molecular Engineering, Peking University, Beijing 100871, China

[2]Beijing National Laboratory for Condensed Matter Physics and Institute of Physics, Chinese Academy of Sciences, Beijing 100190, China

[3]Earth and Environmental Sciences Division and Materials Physics and Applications Division, Los Alamos National Laboratory, Los Alamos, NM 87545, United States

[4]CAS Key Laboratory of Materials for Energy Conversion and State Key Laboratory of High Performance Ceramics and Superfine Microstructure, Shanghai Institute of Ceramics, Chinese Academy of Sciences, Shanghai 200050, China

[5]Collaborative Innovation Center of Quantum Matter, Beijing, China

[*]These authors contribute equally to this work.

Correspondence and requests for materials should be addressed to C.J. (email: Jin@iphy.ac.cn), J.L. (email: jhlin@pku.edu.cn) or F.H. (email: huangfq@pku.edu.cn).

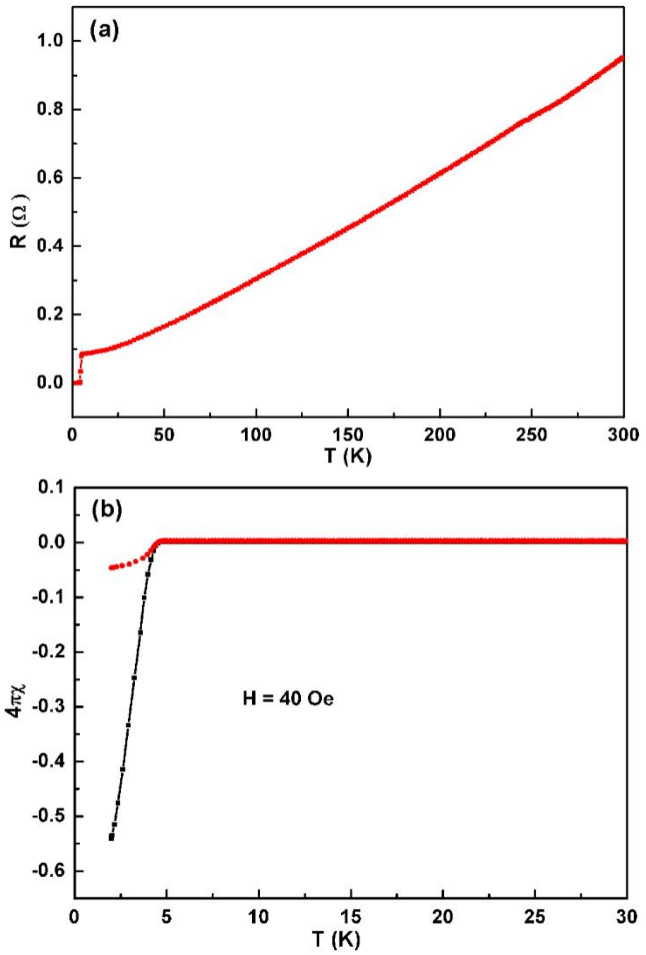

Figure S1 Temperature dependence of (a) the electrical resistance, and (b) the direct-current magnetic susceptibility of tetragonal FeS at ambient pressure.

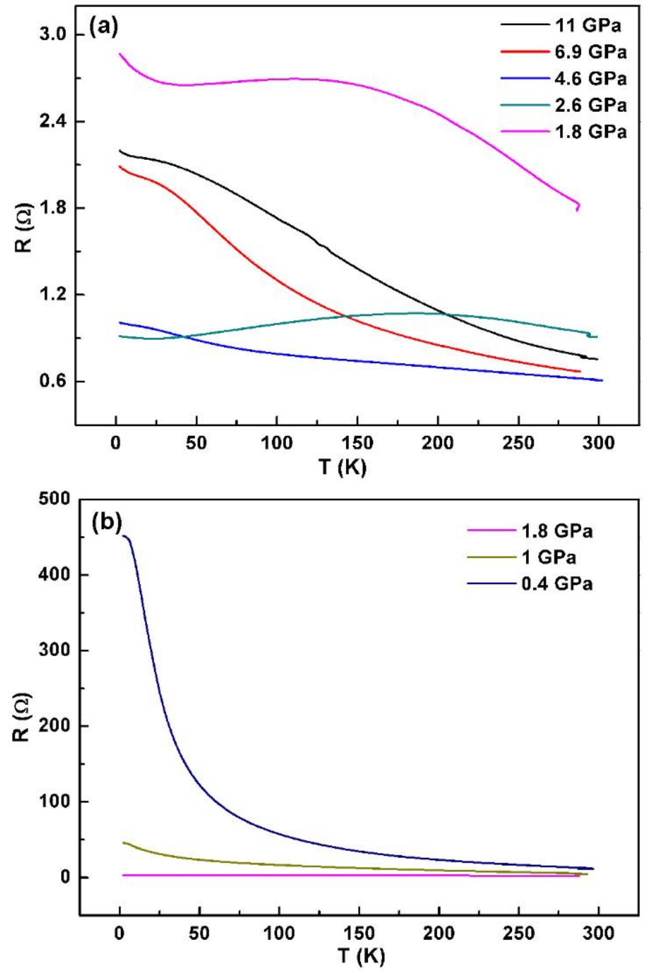

Figure S2 Temperature dependence of resistance for tetragonal FeS at various pressures measured during decompression.

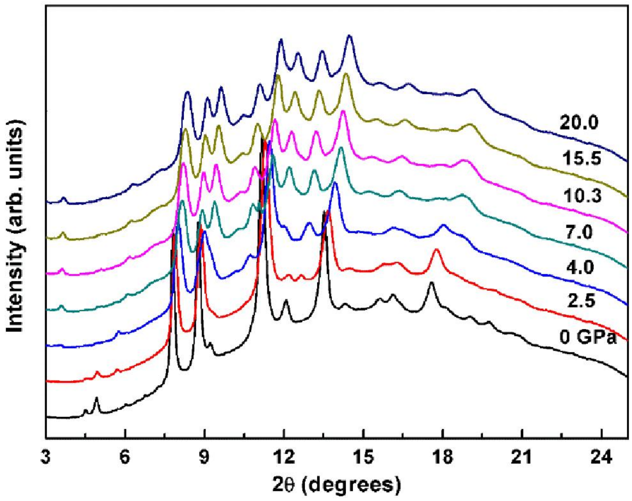

Figure S3 Room-temperature synchrotron X-ray diffraction patterns of tetragonal FeS at various pressures collected during decompression.

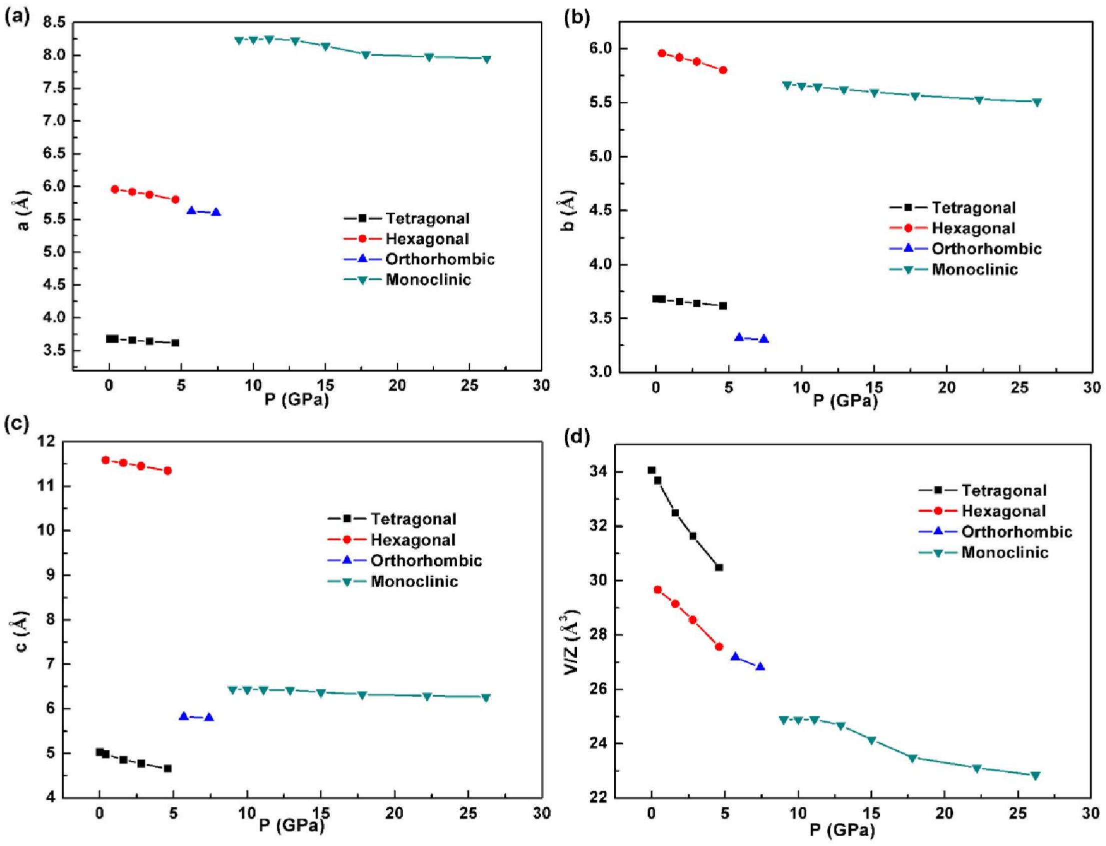

Figure S4 The lattice parameters (a-c) and volume per chemical formula (*V/Z*, *Z* is the number of formula units in the unit cell) (d) of tetragonal FeS for the whole pressure range studied.

Table SI Refined structural parameters for tetragonal FeS obtained from Rietveld refinements of synchrotron XRD data collected at room temperature with increasing pressure.

Pressure	0.4 GPa		7.4 GPa	12.9 GPa
	Tetragonal	Hexagonal	Orthorhombic	Monoclinic
Space group	*P*4/*nmm*	*P*-62*c*	*Pnma*	$P2_1/a$
a (Å)	3.6770(8)	5.95676(4)	5.602(3)	8.229(6)
b (Å)	3.6770(8)	5.95676(4)	3.306(3)	5.623(3)
c (Å)	4.981(2)	11.583769(12)	5.791(5)	6.422(3)
V (Å^3)	67.35(4)	356.0(4)	107.26(14)	296.2(3)
Z	2	12	4	12
R_p	2.54%		2.66%	3.43%
R_{wp}	2.92%		3.41%	4.12%
χ^2	1.52		2.1	2.44
